# Sensitivity of immunochemical faecal occult blood testing for detecting left- *vs* right-sided colorectal neoplasia

**DOI:** 10.1038/bjc.2011.160

**Published:** 2011-05-10

**Authors:** U Haug, K M Kuntz, A B Knudsen, S Hundt, H Brenner

**Affiliations:** 1 Division of Clinical Epidemiology and Aging Research, German Cancer Research Center, Im Neuenheimer Feld 460, Heidelberg 69120, Germany; 2 Division of Health Policy and Management, University of Minnesota, Minneapolis, MN, USA; 3 Department of Radiology, Institute for Technology Assessment, Massachusetts General Hospital, Boston, MA, USA

**Keywords:** colorectal neoplasia, faecal occult blood testing, screening

## Abstract

**Background:**

Faecal occult blood tests (FOBTs) are used for colorectal cancer (CRC) screening. We aimed to assess the sensitivity of an immunochemical FOBT for detecting advanced colorectal neoplasia in the left *vs* the right colon and to explore reasons for potential differences in site-specific test performance.

**Methods:**

We prospectively measured faecal occult blood levels by a quantitative immunochemical FOBT (RIDASCREEN) in 2310 average-risk subjects undergoing screening colonoscopy. We compared diagnostic performance for subjects with left- *vs* right-sided advanced neoplasia, as well as patient characteristics and adenoma characteristics that have been suggested to impact faecal haemoglobin levels.

**Results:**

Sensitivities for subjects with left- *vs* right-sided advanced neoplasia were 33% (95% confidence interval (CI), 26–41%) and 20% (CI, 11–31%) (*P*=0.04) at a specificity of 95% (overall sensitivity: 29%) and the areas under the receiver-operating characteristics curve were 0.71 (CI, 0.69–0.72) and 0.60 (CI, 0.58–0.63), respectively. Pedunculated shape was strikingly more common in participants with left- *vs* right-sided advanced neoplasia (47% *vs* 14%). In logistic regression analyses adjusted for site, pedunculated shape was statistically significantly associated with test sensitivity (*P*=0.04).

**Conclusions:**

The immunochemical FOBT in our study was more sensitive for detecting subjects with left- *vs* right-sided advanced colorectal neoplasia. Our findings may stimulate further diagnostic research in the field as well as modelling analyses to estimate the potential effect of site-specific test performance on the effectiveness of annual or biennial FOBT-based screening programmes, in particular with respect to protection from right-sided CRC.

With >1 million new cases and >500 000 deaths per year worldwide, colorectal cancer (CRC) is the third most common cancer and the fourth most common cancer cause of death globally (Parkin *et al*, 2005). Owing to its slow development from removable precursor lesions (i.e., adenomas) and early cancer stages with good prognosis, screening for CRC has been shown to reduce the mortality of the disease (Mandel *et al*, 1993; Hardcastle *et al*, 1996; Jorgensen *et al*, 2002; Heresbach *et al*, 2006). However, there is a growing body of evidence suggesting that screening colonoscopy, when performed in the community setting, is more effective in protecting from neoplasia in the left colon and rectum than in protecting from neoplasia in the right colon (Brenner *et al*, 2007, 2010b; Lakoff *et al*, 2008; Baxter *et al*, 2009). While different reasons for the poorer protective effect of colonoscopy in the right compared with the left colon are discussed (Ransohoff, 2009), it is an important question whether the sensitivity of the most common non-invasive screening tool for CRC, faecal occult blood testing (FOBT), also differs for left- *vs* right-sided neoplasia. Given that positive FOBT results are typically followed up by colonoscopy, a lower sensitivity both of FOBT and colonoscopy for right-sided neoplasia would make the potential of FOBT screening to protect from right-sided CRC even worse.

From a theoretical point of view, there are at least two arguments that support a higher sensitivity of FOBT for left sided than for right-sided neoplasia. First, degradation of haemoglobin during colon passage could favour the detection of left-sided neoplasia (Rockey, 2010). Second, due to differences in stool consistency, blood may be more homogeneously distributed when originating from the right side and more on the surface when originating from the left side, which would also favour the detection of left-sided neoplasia.

Empirical evidence from screening studies with colonoscopic follow-up of all participants is crucial to precisely estimate site-specific test performance. To date, such evidence is limited. A large study investigated an immunochemical FOBT in >20 000 average-risk subjects and showed a sensitivity of 31 and 16% for left- and right-sided advanced neoplasia, respectively, at a specificity of 95% (Morikawa *et al*, 2005). For guaiac-based FOBT, a similar difference in site-specific test performance was observed by Ahlquist *et al* (2008) in a study with about 2500 average-risk subjects, reporting a sensitivity of 13 and 7% (Hemoccult) and of 27 and 11% (Hemoccult SENSA) for left- and right-sided advanced colorectal neoplasia, respectively. However, neither study provided further information on the subgroups with left- and right-sided advanced neoplasia, and it thus remains unclear whether other factors with potential impact on diagnostic performance of FOBT, such as gender, non-steroidal anti-inflammatory (NSAID) drug use or adenoma characteristics, such as size, pedunculated shape or plural occurrence (Ciatto *et al*, 2007; Morikawa *et al*, 2007; Levi *et al*, 2009; Rozen *et al*, 2009), were differentially distributed between the subgroups.

The aim of this study was to address site-specific performance of a quantitative immunochemical FOBT in a large screening study and to explore factors that could explain potential differences in sensitivity for left- and right-sided advanced neoplasia.

## Materials and methods

### Study design, data and sample collection

We used data from the *B*egleitende Eva*l*uierung *i*nnovativer *T*estverfahren *z*ur Darmkrebsfrüherkennung (BliTz) study to evaluate the site-specific diagnostic performance of an immunochemical FOBT. The BliTz study is an ongoing prospective screening study conducted in southwestern Germany aimed to comparatively evaluate novel tests for early detection of CRC. For the present analyses, we included patients recruited from January 2006 to November 2009. The details of the study design have already been published (Hundt *et al*, 2009; Haug *et al*, 2010; Brenner *et al*, 2010a).

Briefly, the study includes participants of screening colonoscopy – a procedure that the German health care system has offered since October 2002 to average-risk persons 55 years or older. Only experienced endoscopists (internists/gastroenterologists or surgeons with pertinent certified specialisations) having conducted at least 200 colonoscopies and at least 50 polypectomies under supervision in the preceding two calendar years are permitted to conduct screening colonoscopies. Requirements for maintenance of permission include conduction of at least 200 colonoscopies and at least 10 polypectomies per year. Quality control measures also include the image documentation of complete colonoscopy.

Persons undergoing screening colonoscopy typically present at the gastroenterology practice for preliminary consultation about 1 week before colonoscopy. At that time, they were informed about and invited to participate in the study and eligible patients received a study package after informed consent. The study package contained a collection tissue for avoiding contact of the stool with toilet water, a small container and a plastic spoon for stool collection (60 ml), a plastic bag for storage of the container and detailed instructions for stool collection. Stool from one bowel movement was collected at home before bowel preparation for colonoscopy without any specific recommendations for dietary or medicinal restrictions. The stool was collected dry, that is without buffer. Participants were asked to freeze or, if freezing was not possible, to cool the stool sample. Less than 5% of participants reported that freezing was not possible. On the day of colonoscopy, participants rendered the stool-filled container at the gastroenterological practice from where it was shipped on dry ice to a central laboratory and frozen at −20°C until analysis. The study package also contained a standardised questionnaire, which the participants were asked to fill out. Among others, they were asked about current use of specific medication, including analgesics and low-dose aspirin.

After colonoscopy, we collected reports on colonoscopic and histological findings and two independent, trained research assistants extracted information in a standardised manner, while blinded to the results of stool testing. As regards the increasingly recognised subset of serrated polyps (Noffsinger, 2009), these lesions were categorised according to the histological report into adenomas or hyperplastic polyps. Colonoscopies as well as histological examinations were also performed blinded to the results of stool testing.

We thawed the stool-filled containers at a median interval of 4 days after arrival at the central laboratory to perform a quantitative immunochemical FOBT. Faecal haemoglobin levels were measured using an automated ELISA according to the manufacturer's instructions (RIDASCREEN Haemoglobin) (Haug *et al*, 2010). The lower detection limit was 0.42 *μ*g g^–1^ stool. All analyses were done blinded to the results of colonoscopy.

The Ethics committees of the University of Heidelberg and of the physicians’ chambers of Baden–Württemberg, Rheinland Pfalz and Hessen approved the study.

### Statistical analyses

**Figure 1:**
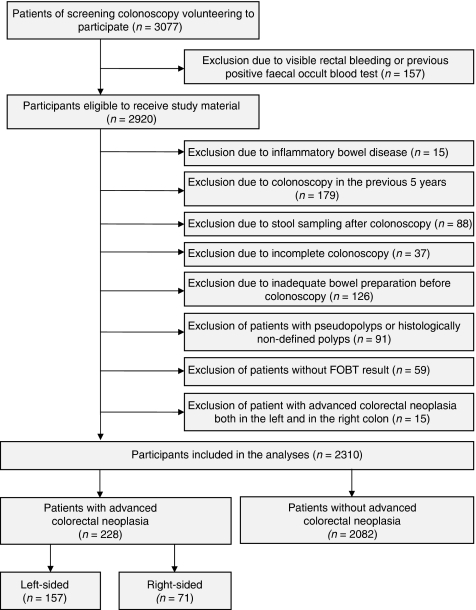
Standards for reporting of diagnostic accuracy (STARD) flow diagram.

As illustrated in Figure 1, we made the following consecutive exclusions to ensure that study participants represent the average-risk target population of CRC screening and to minimise potential misclassification due to missed lesions on colonoscopy: visible rectal bleeding or previous positive FOBT result (*n*=157), inflammatory bowel disease (*n*=15), colonoscopy in the previous 5 years (*n*=179), stool sampling after colonoscopy (*n*=88), incomplete colonoscopy (*n*=37) and inadequate bowel preparation for colonoscopy (*n*=126). In addition, we excluded 91 participants with pseudopolyps or histologically undefined polyps at screening colonoscopy. Another 59 participants had to be excluded due to missing of a suitable stool sample. Among the remaining 2325 participants potentially eligible to be included in the analyses, we excluded 15 participants who were diagnosed with advanced colorectal neoplasia both in the left and in the right colon.

The study population was categorised into the group of subjects with advanced colorectal neoplasia (for calculation of sensitivity) and the group of subjects without advanced colorectal neoplasia (for calculation of specificity). Advanced colorectal neoplasia was defined as CRC or an adenoma with at least one of the following features: size ⩾1 cm, villous components or high-grade dysplasia. For calculation of site-specific sensitivity, we categorised subjects according to the location of the advanced colorectal neoplasia in the right colon (including caecum, ascending colon, right flexure and transverse colon) *vs* the left colon (including left flexure, descending colon, sigmoid colon and rectum). To assess whether there is a progressive increase in site-specific sensitivity going from the right to the left side of the colon, we also calculated sensitivities for a more detailed stratification according to anatomical site.

We calculated sensitivities for left- and right-sided advanced colorectal neoplasia and specificity at different cutoff levels (i.e., different threshold levels for test positivity) and calculated the corresponding 95% confidence intervals (CIs) based on the exact binomial distribution. We assessed the statistical significance of differences in sensitivities between subjects with right- and left-sided advanced colorectal neoplasia at clinically relevant cutoff levels (i.e., cutoff levels yielding specificities that are typically required in the screening setting) using two-sided *χ*^2^ tests with an *α* level of 0.05.

To illustrate the discriminatory power of the test for advanced colorectal neoplasia stratified by anatomical site (right- *vs* left-sided), we constructed receiver-operating characteristic (ROC) curves by plotting site-specific true positive rates (i.e., site-specific sensitivities) against the corresponding false positive rates (i.e., 100%−specificity) for different cutoff levels. Each point on an ROC curve thus represents a sensitivity/specificity pair corresponding to a particular positivity threshold. We calculated the areas under the ROC curves (AUCs) and the corresponding 95% CIs using the method of DeLong *et al* (1988). The AUC is a measure of how well a quantitative test can distinguish between subjects with and without a disease.

To explore whether potential differences in site-specific test performance may be due to an unequal distribution of other factors that have been suggested to impact faecal haemoglobin levels, such as sex, age, current NSAID use and the size and shape of colorectal neoplasia (Ciatto *et al*, 2007; Morikawa *et al*, 2007; Levi *et al*, 2009; Rozen *et al*, 2009), we compared the subgroups with left- and right-sided advanced colorectal neoplasia regarding these factors. Current NSAID use refers to individuals who reported in the questionnaire that they are currently taking analgesic or low doses of aspirin or other NSAIDs. We compared median faecal haemoglobin levels for factors that could explain a higher sensitivity for left-sided neoplasia according to their distribution by site and used logistic regression analyses to assess their association with the test sensitivity adjusted for site.

To explore further reasons that could result in different diagnostic performance for left- and right-sided neoplasia, we conducted sensitivity analyses, where we subset on individuals with exactly one advanced colorectal neoplasm in either the left or right colon and no other adenomas. This restriction removed a potential source of heterogeneity between the subgroups with left- and right-sided advanced neoplasia – other adenomas. Excluding subjects with multiple adenomas eliminated interfering effects that could result from more than one adenoma in the same individual (such as a higher likelihood of bleeding due to plural occurrence of adenomas). We constructed ROC curves stratified by anatomical site and calculated AUCs and corresponding 95% CIs including (1) only cases with one advanced colorectal neoplasm, (2) only cases with one advanced adenoma (i.e., no CRC cases) and (3) only cases with one large adenoma (⩾1 cm).

In additional sensitivity analyses, we focused on subjects with right-sided advanced colorectal neoplasia who did not have any lesion in the left colon and we assessed whether there was a difference in test performance in this restricted group compared with the whole group of subjects with right-sided advanced colorectal neoplasia.

We used MedCalc for Windows, version 9.6.4.0 (MedCalc Software, Mariakerke, Belgium) for the ROC curves analyses and SAS version 9.1 (SAS Institute Inc., Cary, NC) for all other statistical analyses.

## Results

Overall, 2310 study participants were included in the analysis. There were 157 and 71 subjects with advanced colorectal neoplasia in the left and the right colon, respectively, and 2082 subjects without advanced colorectal neoplasia (Figure 1).

Table 1 shows site-specific test performance of the immunochemical FOBT at cutoff levels of 2, 4, 8 and 15 haemoglobin per g stool, yielding specificities between 88% and 97%. At these levels of specificity, sensitivity for left-sided advanced colorectal neoplasia was 12–13 percentage points higher than for right-sided advanced neoplasia. For example, at a specificity of 95%, corresponding with a cutoff level of 8 *μ*g haemoglobin per g stool, sensitivity (95% CI) for subjects with left- and right-sided advanced neoplasia was 33% (26–41%) and 20% (11–31%), respectively (*P*=0.04). A more detailed anatomical stratification into caecum, ascending colon, right to left flexure, descending to sigmoid colon and rectum showed sensitivities (95% CI) of 13% (2–40%), 19% (6–38%), 26% (9–51%), 35% (25–46%) and 23% (12–36%) at a specificity of 95%.

**Figure 2:**
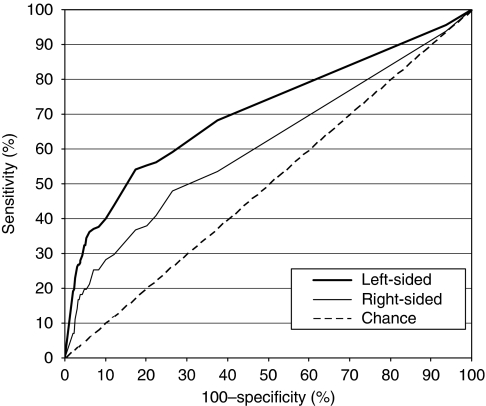
ROC curves for detecting patients with advanced colorectal neoplasia stratified by anatomical subsite, using a quantitative immunochemical FOBT. (In an ROC curve, the true positive rate (sensitivity) is plotted in function of the false positive rate (100−specificity) for different positivity thresholds (i.e., different cutoff levels) of a quantitative test (here, faecal haemoglobin levels). The AUC is a measure of how well a quantitative test can distinguish between subjects with and without a disease.)

Receiver-operating characteristic curves analysis showed that sensitivities for detecting subjects with advanced neoplasia in the left colon were higher compared with the right colon over the whole range of specificities, that is at all cutoff levels (Figure 2). The AUCs (95% CIs) were 0.71 (0.69–0.72) for the subgroup with advanced neoplasia in the left colon and 0.60 (0.58–0.63) for the subgroup with advanced neoplasia in the right colon. The difference in site-specific AUCs did not change when CRC cases were excluded from the analysis (Table 2).

A comparison of the diagnostic subgroups regarding factors that might impact faecal haemoglobin levels is shown in Table 3. The mean and median age was 65 years in participants with left- and right-sided advanced neoplasia. The proportion of male participants and the proportion of current NSAID users were slightly higher in the subgroup with right-sided advanced neoplasia than in the subgroup with left-sided advanced neoplasia. The proportion of subjects with large neoplasia (⩾1 cm in diameter), the proportion of subjects with more than one neoplasm, as well as the proportion of subjects with more than one advanced neoplasm was also higher in the subgroup with right-sided advanced neoplasia. In contrast, the proportion of subjects with pedunculated adenomas was markedly higher in subjects with left- *vs* right-sided advanced neoplasia (41 out of 88, corresponding to a proportion of 0.47, *vs* 6 out of 42, corresponding to a proportion of 0.14). The median (interquartile range) faecal haemoglobin levels were 3.3 (0.4–41.0) *μ*g g^−1^ in the 47 subjects with pedunculated adenomas compared with 0.4 (<0.4–7.4) in the 83 subjects with otherwise shaped adenomas, with the difference being statistically significant (*P*=0.02). Logistic regression analyses adjusted for site showed a statistically significant association of pedunculated shape with test sensitivity (*P*=0.04).

**Figure 3:**
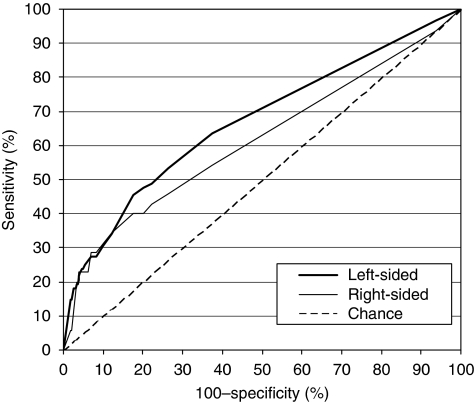
ROC curves for detecting patients with one advanced colorectal neoplasm (and no other colorectal adenomas) stratified by anatomical subsite, using a quantitative immunochemical FOBT. (In an ROC curve, the true positive rate (sensitivity) is plotted in function of the false positive rate (100−specificity) for different positivity thresholds (i.e., different cutoff levels) of a quantitative test (here, faecal haemoglobin levels). The AUC is a measure of how well a quantitative test can distinguish between subjects with and without a disease.)

Sensitivity analyses focusing on subjects with only one advanced neoplasm (and no other adenomas) included 88 subjects with a left-sided neoplasm and 35 subjects with a right-sided neoplasm. The distribution of factors that may impact faecal haemoglobin levels in this restricted study population was similar to the distribution observed in the main analysis (Appendix). Figure 3 illustrates the ROC curves for detecting subjects with one advanced colorectal neoplasm (and no other adenomas), stratified by left- and right-sided location. While there was no clear difference at higher levels of specificity, the ROC curves for right- and left-sided advanced neoplasia diverged (towards higher sensitivities for left-sided advanced neoplasia) starting at a specificity of about 87%, which corresponds with a cutoff level of 2 *μ*g haemoglobin per g stool. Again, the AUC was larger for the subgroup with one left-sided advanced neoplasm than for the subgroup with one right-sided advanced neoplasm and the difference persisted when further exclusion criteria were applied to decrease heterogeneity between subgroups with respect to the type and size of the advanced neoplasm (Table 2).

In the subgroup of the 60 subjects with right-sided advanced colorectal neoplasia who did not have any lesion in the left colon, there was no difference in test performance compared with the whole group of subjects with right-sided advanced colorectal neoplasia (*N*=71). Sensitivities were 22 and 20% in the former and the latter group at a cutoff level of 8 *μ*g g^–1^, and the AUCs were 0.60 and 0.61, respectively.

## Discussion

We extensively addressed the question of whether FOBT, the most common non-invasive tool for CRC screening, shows differential sensitivity for detecting left- *vs* right-sided advanced colorectal neoplasia within a large screening study conducted in average-risk subjects. Our analysis of data on an ELISA-based immunochemical FOBT supports the hypothesis that FOBT is more sensitive for detecting left-sided advanced neoplasia than right-sided advanced neoplasia. The magnitude of the difference in sensitivities (about 13 percentage points) at a specificity of 95% is similar to the findings reported by Morikawa *et al* (2005) regarding an agglutination-based immunochemical FOBT (11) and is also supported by another studies reporting on site-specific test performance of a guaiac-based FOBT (Ahlquist *et al*, 2008).

However, the primary focus of the aforementioned studies was on overall test performance; the authors did not provide further information on the characteristics of the subgroups with left- and right-sided advanced neoplasia. We focused, for the first time, on this stratification according to anatomical site and explored potential factors that could explain a higher sensitivity of FOBT for left-sided neoplasia.

Specifically, we described the subgroups with left- and right-sided advanced neoplasia with respect to parameters that were reported to correlate with a higher sensitivity of FOBT, such as male gender, current NSAID use and adenoma characteristics (Ciatto *et al*, 2007; Morikawa *et al*, 2007; Levi *et al*, 2009; Rozen *et al*, 2009). The proportion of men, the proportion of current NSAID users, the proportion of subjects with large adenomas (⩾1 cm in diameter) as well as the proportion of subjects with more than one adenoma was higher in the subgroup with right-sided advanced neoplasia than in the subgroup with left-sided advanced neoplasia. Thus, the distribution of these parameters could not explain the observation of a higher sensitivity for left-sided advanced neoplasia. In contrast, the proportion of subjects with pedunculated adenomas was strikingly higher in the subgroup with left-sided advanced neoplasia (0.47 *vs* 0.14). Although information on adenoma shape was often missing, it is unlikely that this difference is caused by information bias. The latter would have occurred if the likelihood of reporting on pedunculated shape had been differential with respect to the location of adenomas, which doesn’t seem plausible. The proportion of participants for whom information on adenoma shape was missing altogether was the same in the subgroup with left- and right-sided advanced neoplasia. The higher proportion of pedunculated adenomas in the left colon is consistent with autopsy studies that reported on the shape of adenomas according to anatomical site (Blatt, 1961; Eide and Stalsberg, 1978; Rickert *et al*, 1979; Williams *et al*, 1982). The comparison of median faecal haemoglobin levels in subjects with pedunculated adenomas *vs* subjects with otherwise shaped adenomas as well as logistic regression analyses adjusted for site supported the importance of pedunculated shape regarding the site difference in test sensitivity. The proportion of CRC cases was also higher in the subgroup with left-sided neoplasia, but the respective numbers were small and their exclusion did not change the observed differences in site-specific test performance.

In the primary analysis of ROC curves, we observed a higher sensitivity for individuals with left-sided advanced neoplasia at all cutoff levels, including cutoff levels yielding specificities that are typically required in the screening setting (i.e., well above 90%). In sensitivity analysis, we explored whether the difference in sensitivities according to anatomical site was due to the location of the advanced neoplasm itself. For that purpose, we restricted the analyses of ROC curves to individuals with one advanced colorectal neoplasm and no other adenomas. Even though this restriction created an artificial setting that does not reflect the distribution of neoplasms in the natural setting, it avoided interfering effects that could result from more than one adenoma in the same individual, which are potentially distributed over different anatomical sites. Interestingly, the difference in diagnostic performance according to anatomical site in this restricted setting occurred only when sensitivity was increased by shifting the cutoff level to lower levels (i.e., lower specificity), but was not observed at higher, clinically relevant cutoff levels (i.e., at levels yielding specificities well above 90%). This finding could be interpreted as follows: when the cutoff level is high, which means that only individuals with a relatively strong source of bleeding test positive, it doesn’t matter whether the advanced neoplasm is in the right or in the left colon. When the cutoff level is lowered, which allows individuals with a weaker source of bleeding to test positive as well, then sensitivity is higher for left-sided than for right-sided advanced neoplasia. In other words, our results suggest that weak sources of bleeding are more likely to occur (possibly due to pedunculated shape of adenomas) or to be detected (possibly due to degradation of haemoglobin) in the left colon than in the right colon, while the location doesn’t matter when there is a strong source of bleeding.

An explanatory model that synthesises the different findings could be as follows: a certain proportion of advanced neoplasms present by themselves a relatively strong source of bleeding and their likelihood of being detected through FOBT does not depend on the anatomical site. Another proportion of advanced neoplasms is weak sources of bleeding and do not lead by themselves to a positive FOBT at clinically relevant cutoff levels. However, in conjunction with other adenomas, which may also represent weak sources of bleeding, haemoglobin levels may mount up leading to a positive FOBT at clinically relevant cutoff levels (i.e., at levels yielding specificities well above 90%). This additive mechanism may cause the higher sensitivity for left-sided neoplasia at clinically relevant cutoff levels since our findings suggested that the detection (and/or occurrence) of weak sources of bleeding is more likely in the left colon than in the right colon.

Irrespective of the reasons explaining the higher sensitivity of FOBT for left-sided advanced neoplasia, the finding itself would be of clinical relevance. While colonoscopy has already been questioned regarding protection from right-sided colorectal neoplasia (Brenner *et al*, 2007, 2010b; Lakoff *et al*, 2008; Baxter *et al*, 2009), our study supports the hypothesis that FOBT, the most common non-invasive tool for CRC screening, also shows lower performance for right-sided neoplasia. Our findings may stimulate further diagnostic research in the field. They may also provide valuable information for modelling analyses to estimate the potential effects of site-specific test performance on the programmatic sensitivity and the effectiveness of FOBT-based screening programmes in which FOBT is repeated at frequent intervals (e.g., annually or biennially). In particular, current strategies that combine flexible sigmoidoscopy and FOBT could be affected by a lower sensitivity of FOBT for right-sided lesions (Pignone *et al*, 2002; Zauber *et al*, 2008). Such strategies have been suggested to be as effective as colonoscopy in some scenarios, but those findings might change if site-specific test performance of FOBT is taken into account. Generally, site-specific performance should be a focus in the optimisation of current screening tests as well as in the development and evaluation of novel screening tests, such as computed tomographic colonography (Heresbach *et al*, 2011) and colon capsule endoscopy (Sacher-Huvelin *et al*, 2010).

Our study offered good opportunity for evaluating site-specific test performance due to the fact that all study participants underwent colonoscopy irrespective of the FOBT result and due to a setting that reflects the average-risk target population of CRC screening. Using a symptomatic study population or using a clinical follow-up instead of colonoscopy to estimate diagnostic performance could bias site-specific estimates of test sensitivity since the presence of symptoms or the likelihood of clinical manifestation may depend on anatomical site. The prospective design, the careful application of exclusion criteria as well as the high level of experience and qualification among gastroenterologist who participate in the German screening colonoscopy programme were favourable in terms of minimising potential sources of bias.

Our study has also important limitations. First, the group with ‘advanced neoplasia’ was comprised for the most part individuals with advanced adenomas, while the number of individuals with CRC was low. Given that advanced adenomas are considered to be precursors to CRC, our results thus support site-specific effects with respect to prevention from CRC through FOBT. To investigate whether there are also site-specific effects with respect to early detection of invasive CRC, very large sample sizes would be needed since the prevalence of undetected CRC is inherently low in an average-risk study population. From a theoretical point of view, site-specific effects might be less important for invasive CRCs, which, if they bleed, have been reported to go along with comparatively high faecal haemoglobin levels (Levi *et al*, 2007). Second, although we had a reasonable sample size of participants with advanced neoplasia, further stratification according to parameters that have been suggested to impact faecal haemoglobin levels and calculation of site-specific test performance for each of the subgroups would not have been possible due to sample size limitations. We, therefore, only assessed whether these parameters were differentially distributed across the subgroups with left- and right-sided advanced colorectal neoplasia. There were also sample size limitations regarding the more detailed stratification of sensitivities according to anatomical site. Although the point estimates of these analyses tentatively suggest a progressive increase in sensitivity from the right to the left side of the colon (excluding the rectum), the CIs of these estimates were large. The comparatively low sensitivity for rectal lesions could be due to the exclusion criterion ‘visual bleeding’, which may apply more often to subjects with rectal neoplasms that bleed than to subjects with neoplasms at other parts of the colon (where it may be less likely that the blood is detected visually). Third, it would be interesting to investigate whether site-specific test performance is affected by the number of stool samples that are tested per person or by different sampling techniques, which, however, we could not assess in our study.

We did not consider site-specific performance of FOBT with respect to any adenoma, but focused on advanced adenomas because they are considered to be the most clinically relevant precursor lesions. This focus also minimised potential misclassification bias due to miss rates at colonoscopy, which may be site specific, but which have been shown to be generally low for advanced neoplasia (van Rijn *et al*, 2006; Heresbach *et al*, 2008).

In conclusion, the immunochemical FOBT in our study was more sensitive for detecting subjects with left- *vs* right-sided advanced colorectal neoplasia. Our findings may stimulate further research in the field as well as modelling analyses to estimate the potential effect of site-specific test performance on the programmatic sensitivity and the effectiveness of annual or biennial FOBT-based screening programmes, in particular with respect to protection from right-sided CRC.

## Figures and Tables

**Table 1:** Sensitivity and specificity of the immunochemical faecal occult blood test for detecting individuals with left- *vs* right-sided advanced colorectal neoplasia at different cutoff levels

**Cutoff level (*μ*g g^–1^)**	**Specificity (95% confidence interval)**	**Sensitivity (95% confidence interval)**		***P*-value (left-*vs* right- sided)**
		**Left-sided**	**Right-sided^a^**	
15	97% (96–98%)	26% (19–34%)	14% (7–24%)	0.04
8^a^	95% (94–96%)	33% (26–41%)	20% (11–31%)	0.04
4	92% (90–93%)	38% (30–46%)	25% (16–37%)	0.07
2	88% (86–89%)	44% (36–52%)	30% (19–42%)	0.04

a As described in Results section, sensitivity in this subgroup did not change when restricting the analyses to the 60 subjects with right-sided advanced colorectal neoplasia, who did not have any lesion in the left colon.

**Table 2:** Areas under the ROC) curves^a^ of the immunochemical faecal occult blood test for detecting left- *vs* right-sided advanced colorectal neoplasia

**Diagnostic subgroup**	**Area under the ROC curve^a^ (95% confidence interval)**	
	**Left-sided**	**Right-sided^b^**
Advanced neoplasm(s)	0.71 (0.69–0.72)	0.60 (0.58–0.63)
Advanced adenoma(s) (no CRC)	0.69 (0.67–0.71)	0.60 (0.58–0.62)
		
*Sensitivity analyses* c		
One advanced neoplasm	0.67 (0.65–0.69)	0.61 (0.59–0.63)
One advanced adenoma (no CRC)	0.66 (0.64–0.68)	0.60 (0.58–0.62)
One large adenoma (⩾1 cm)	0.71 (0.69–0.73)	0.66 (0.64–0.68)

Abbreviations: AUC=area under the ROC curve; CRC=colorectal cancer; ROC=receiver-operating characteristics.

a In an ROC curve, the true positive rate (sensitivity) is plotted in function of the false positive rate (100−specificity) for different positivity thresholds (i.e., different cutoff levels) of a quantitative test (here, faecal haemoglobin levels). The AUC is a measure of how well a quantitative test can distinguish between subjects with and without a disease.

b As described in Results section, sensitivity in this subgroup did not change when restricting the analyses to the 60 subjects with right-sided advanced colorectal neoplasia who did not have any lesion in the left colon.

c Only individuals with one advanced colorectal neoplasm (and no other adenomas) are included here as described in Materials and Methods section.

**Table 3:** Comparison of the diagnostic subgroups with respect to factors that have been suggested to potentially impact faecal haemoglobin levels

	**Subjects with advanced colorectal neoplasia** a		
	**Left-sided (*N*=157)**	**Right-sided (*N*=71)**	**Subjects without advanced colorectal neoplasia (*N*=2082)**
Mean age (years)	65	65	62
Median age (years) (interquartile range)	65 (59–70)	65 (59–69)	61 (57–67)
Proportion men	0.62	0.65	0.48
Proportion current NSAID users^b^	0.12	0.17	0.15
Proportion subjects with neoplasm(s) ⩾1 cm	0.68	0.76	N.a.
Proportion subjects with more than one neoplasm	0.44	0.51	N.a.
Proportion subjects with more than one advanced neoplasm	0.12	0.14	N.a.
Proportion subjects with pedunculated adenoma(s)^c^	0.47	0.14	N.a.
Proportion with CRC	0.08	0.01	N.a.

Abbreviations: CRC=colorectal cancer; N.a.=not applicable; NSAID=non-steroidal anti-inflammatory drugs.

a Defined as CRC or adenoma ⩾1 cm in size, adenomas with villous components or adenomas with high-grade dysplasia.

b As described in Materials and Methods section, this variable also includes users of low-dose aspirin.

c We only considered subjects for whom information on shape was reported for all adenomas that were detected at colonoscopy; the percentages thus refer to 88 subjects with left-sided neoplasm(s) and 42 subjects with right-sided neoplasm(s).

**Table A1:** Comparison of the diagnostic subgroups with respect to factors that have been suggested to potentially impact faecal haemoglobin levels for subjects who were included in the sensitivity analyses

	**Subjects with one advanced colorectal neoplasm^a^**		
	**Left-sided (*N*=88)**	**Right-sided (*N*=35)**	**Subjects without advanced colorectal neoplasia (*N*=2082)**
Mean age (years)	64	65	62
Median age (years) (interquartile range)	64 (58–69)	65 (59–70)	61 (57–67)
Proportion men	0.56	0.63	0.48
Proportion current NSAID users^b^	0.11	0.29	0.15
Proportion subjects with neoplasm(s) ⩾1 cm	0.60	0.77	N.a.
Proportion subjects with more than one neoplasm	N.a.	N.a.	N.a.
Proportion subjects with more than one advanced neoplasm	N.a.	N.a.	N.a.
Proportion subjects with pedunculated adenoma(s)^c^	0.48	0.07	N.a.
Proportion with CRC	0.06	0.03	N.a.

Abbreviations: CRC=colorectal cancer; N.a.=not applicable; NSAID=non-steroidal anti-inflammatory drugs.

a Defined as CRC or adenoma ⩾1 cm in size, adenomas with villous components or adenomas with high-grade dysplasia; only individuals with one advanced colorectal neoplasm (and no other adenomas) are included here as described in Materials and Methods section.

b As described in Materials and Methods section, this variable also includes users of low-dose aspirin.

c Information on shape was reported for 62 subjects with a left-sided neoplasm and 27 subjects with a right-sided neoplasm. The percentages refer to these subjects.

## Appendix

Table A1
